# Structural Studies of Predicted Ligand Binding Sites and Molecular Docking Analysis of *Slc2a4* as a Therapeutic Target for the Treatment of Cancer

**DOI:** 10.3390/ijms19020386

**Published:** 2018-01-28

**Authors:** Raphael Taiwo Aruleba, Tayo Alex Adekiya, Babatunji Emmanuel Oyinloye, Abidemi Paul Kappo

**Affiliations:** 1 Biotechnology and Structural Biochemistry (BSB) Group, Department of Biochemistry and Microbiology, University of Zululand, KwaDlangezwa 3886, South Africa; arulebataiwo@yahoo.com (R.T.A.); adekiyatalex@gmail.com (T.A.A.); KappoA@unizulu.ac.za (A.P.K.); 2 Department of Biochemistry, Afe Babalola University, PMB 5454, Ado-Ekiti 360001, Nigeria

**Keywords:** antimicrobial peptides, cancer, docking, homology modelling, *Slc2a4*

## Abstract

Presently, many studies have focused on exploring in silico approaches in the identification and development of alternative therapy for the treatment and management of cancer. Solute carrier family-2-member-4-gene (*Slc2a4*) which encodes glucose transporter 4 protein (GLUT4), has been identified as a promising therapeutic target for cancer. Though *Slc2a4* is known to play a major regulatory role in the pathophysiology of type 2 diabetes, emerging evidence suggests that successful pharmacological inhibition of this protein may lead to the development of a novel drug candidate for the treatment of cancer. In this study, *Slc2a4* protein sequence was retrieved and analysed using in silico approaches, and we identified seven putative antimicrobial peptides (AMPs; RAB1-RAB7) as anti-cancer. The structures of the protein and AMPs were modelled using I-TASSER server, and the overall quality of the *Slc2a4* model was validated using PROCHECK. Subsequently, the probable motifs and active site of the protein were forecasted. Also, the molecular interaction between the AMPs and *Slc2a4* was ascertained using PatchDock. The result revealed that, all the AMPs are good *Slc2a4* inhibitors with RAB1 having the highest binding affinity of 12,392 and binding energy of −39.13 kcal/mol. Hence, this study reveals that all the generated AMPs can serve as therapeutic drug in treating cancer by inhibiting *Slc2a4* which is responsible for the production of energy for cancer cells during angiogenesis. This is the first report on AMPs as inhibitors of *Slc2a4* for the treatment of cancer.

## 1. Introduction

Over the last several decades, there has been a fundamental shift in the global disease burden from infectious diseases to cancer and other non-communicable diseases [1]. Cancer is a group of multifactorial disease caused by the uncontrolled growth and spread of abnormal cells in the body. Cancer cells are characterized by their ability to rapidly grow and divide, and undergo uncontrolled proliferation [2,3]. Cancer is a major public health issue and has been implicated as a global menace exacerbated by environmental and lifestyle factors [4]. It is believed that this group of diseases are associated with both genetic and metabolic abnormalities [5]. However, accumulating evidence indicates that cancer is predominantly a metabolic disease. This fact is in agreement with the original theory of Otto Warburg [6].

The burden of this disease is predicted to increase worldwide due to recent advancement and aging of the population, mainly in the developing countries, where about 82% of the world’s population resides [7]. According to GLOBOCAN estimates in 2012, about 14.1 million new cancer cases were diagnosed in 2012, with cancer accounting for about 8.2 million deaths globally in the same year. Except preventive and adequate management measures are put in place, global cancer death rate has been projected to rise to about 14.6 million annually by 2035 [7,8,9]. Although great strides with significant results have been made in the treatment and control of cancer progression, noteworthy severe side effects on normal tissues and organs combined with various deficiencies of the current chemotherapeutic drugs used in the treatment cancer calls for urgent attention and development of an alternative therapy for the treatment and control of cancer [10,11].

It has been reported in literature that due to the requirement of energy to feed and maintain survival of uncontrolled proliferation, cancer cells consume more glucose than their normal counterparts [2]. This unique attribute of cancer cells (elevated glucose utilization) is been exploited for the development of novel therapy and diagnostic tools for cancers. Assessment of glucose entry into tum our cells establishes glucose uptake to be a key rate-limiting step in glucose metabolism, suggesting the efficacy of targeting glucose transport for therapy [12]. Emerging evidence suggests that pharmacological inhibition of Solute carrier family-2-member-4-gene (*Slc2a4*), which encodes glucose transporter 4 protein (GLUT4), is an attractive therapeutic target for the development of a novel drug candidate for the treatment of cancer [12,13,14]. Members of this family have tissue specific expression, biochemical properties and physiologic functions that operate together to regulate and maintain glucose levels and distribution. *Slc2a4* is an insulin-sensitive glucose transporter known to play an essential role in glucose homeostasis [15].

It has been established that increased cellular glucose uptake and metabolism is a vital requirement for rapid proliferation in tumorigenesis [14]. GLUT4 demonstrates a unique relationship with cancer in a manner comparable to that of GLUT1, since both transporters are transcriptionally repressed by p53, a known tumour suppressor protein that plays an essential role in cell cycle control and apoptosis [16]. Any alteration or mutation within the DNA-binding domain of p53 will possibly lead to dysregulation or overexpression of GLUT4 in certain types of cancer. This important factor makes this protein an attractive target in treatment of cancer [14,16].

Antimicrobial peptides (AMPs) are currently been explored as an essential source for the development of new therapeutic drugs due to their multifunctional properties. They can act as drug delivery vector, signalling molecule, immunomodulatory agent, and mitogenic and antitumour agent [17]. This study using in silico approach sought to identify potential plant antimicrobial peptides as selective inhibitors of *Slc2a4* in order to develop a more potent anti-cancer therapy with high efficacy, excellent tolerability, and few transient side effects.

## 2. Results

### 2.1. Physicochemical Properties and the Abundance of Amino Acids in Slc2a4 Protein

**Figure 1:**
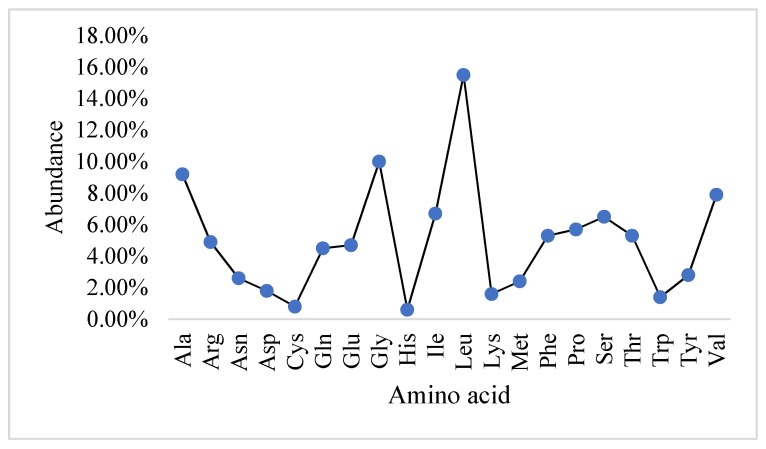
Graphical representation of the abundance of 20 amino acid presents in *Slc2a4*. Leucine has the highest abundance, and histidine has the lowest abundance.

The ExPASy result indicated that *Slc2a4* protein sequence comprises of 509 amino acid residues with all the 20 amino acids (Figure 1) contributing to give the protein an average molecular weight of 54.8 kDa. Additionally, Figure 1 indicated that the most abundant amino acids in *Slc2a4* is leucine with 15.5%, followed by glycine, alanine, and valine with the percentage of 10%, 9.2%, and 7.9%, respectively. Histidine and cysteine contributed the lowest abundance residues with 0.6% and 0.8% respectively, followed by tryptophan, lysine, and aspartate, which contributed 1.4%, 1.6%, and 1.8% amino acid residue to *Slc2a4*, respectively.

In Table 1, the physicochemical parameters predicted the protein to be a neutrally charged protein as a result of equal numbers of positively charged residues (arginine 4.9% and lysine 1.6%) and negatively charged residues (aspartic acid 1.8% and glutamic acids 4.7%), respectively. These four amino acids are the major contributors to the overall charge of a protein because at neutral pH, arginine and lysine are positively charged, while aspartate and glutamate are negatively charged [18].

Moreover, with 2530 Carbon (C), 4009 Hydrogen (H), 641 Nitrogen (N), and 686 Oxygen (O), the atomic composition of *Slc2a4* adds up to 7882. Also, the protein is acidic as indicated by the isoelectric point which was 6.86. The estimated half-life of this protein shows that the protein can remain intact without being degraded for 30 h in human, less than 20 h in yeast and less than 10 h in *E. coli*. The extinction coefficient which describes the quantity of light a protein can absorb at a specific wavelength was computed using tyrosine, tryptophan and cysteine. The generated aliphatic index was 118.61, with 0.556 GRAVY and instability index of 39.83.

### 2.2. Prediction of 2D-Structure of Slc2a4 Protein

**Figure 2:**
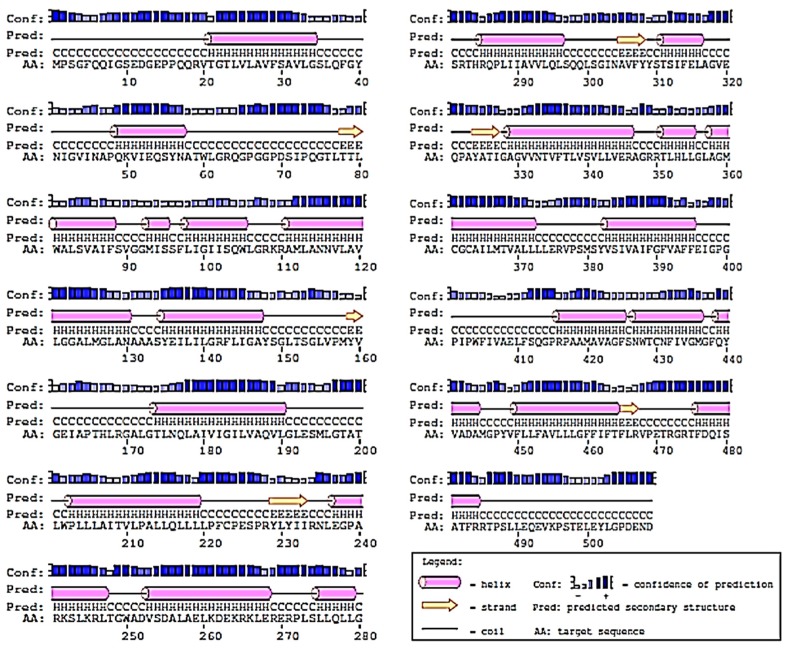
Secondary structure prediction of *Slc2a4* using PSIPRED. *Slc2a4* is predicted to consist of 22 α-helices and 6 β-strands.

As presented in Figure 2, the predicted result by PsiPred server revealed that the secondary structure of *Slc2a4* contains 22 α-helices, 6 β-strands, and 28 coils. The high number of α-helices in the predicted 2D structure confirmed that *Slc2a4* is a transmembrane protein, capable of mediating the transportation of glucose across the cell membrane [19,20,21]. A lot of transmembrane proteins comprise exclusively of α-helices that are available in the cytoplasmic membrane region, while some membrane proteins do have β-strands [22].

### 2.3. Discovery of Putative AMPs and Physicochemical Characterization

Antimicrobial Peptide Databases (CAMP and DRAMP) were visited to retrieve experimentally validated anticancer AMPs. It was revealed that CAMP and DRAMP had 28 and 277, respectively, which are experimentally validated, interred, and synthetic anticancer AMPs. Only 25 anticancer AMPs that have been experimentally validated from plants origin were identified and retrieved. Moreover, the retrieved data was suggested to further analysis in order to remove the experimentally validated anticancer AMPs that are duplicated, and finally only 13 AMPs were generated.

The HMMER multiple module was used in the construction of the AMPs profile, and the ENSEMBL server (http://www.ensembl.org/index.html) and UNIPROT database (http://www.uniprot.org/) were visited to retrieve at least a thousand genome sequences in FASTA format. In order to identify the putative anti-cancer AMPs, the constructed profile was queried against all the genome sequences with an E-value cut-off of 0.01. Seven (7) peptides were identified which were all considered to be putative anticancer AMPs and all identified AMPs were ranked in accordance to their E-values, starting from the lowest to the highest.

Thereafter, the physicochemical properties of the seven generated best putative anticancer AMPs were determined to ascertain the characteristics embedded in them for further usage in interaction study with *Slc2a4*. As shown in Table 2, it was discovered that none of the putative AMPs has 100% similarity with the occurring anticancer AMPs or experimentally validated AMPs, which indicated that all the seven identified sequence of the putative AMPs were novel. Also, it was discovered that out of the seven putative anticancer AMPs generated, two of the AMPs had positive net charge and another two of the AMPs has net negative charge, while three has zero net charge; this is zero net charge may be due to the low or absent of positively charged amino acids in the AMP sequence. Furthermore, the physicochemical analysis of the putative peptide indicates that most of the peptide contain higher amount of cysteine residues, which indicated that the homologue 3D model of the peptides will be properly folded, and this will also contribute to the binding affinity of the peptides to the *Slc2a4*.

Moreover, it was ascertained that all the putative anticancer peptides have hydrophobic values that are above 30%, which was the anticipated value for hydrophobicity content of an antimicrobial peptide, and this may pose a significant impact on the binding affinity of the putative AMPs to their cells and receptors [23,24].

### 2.4. Predicted 3D Homology Model of the Anti-Cancer Putative AMPs and Slc2a4 by I-TASSER

**Figure 3:**
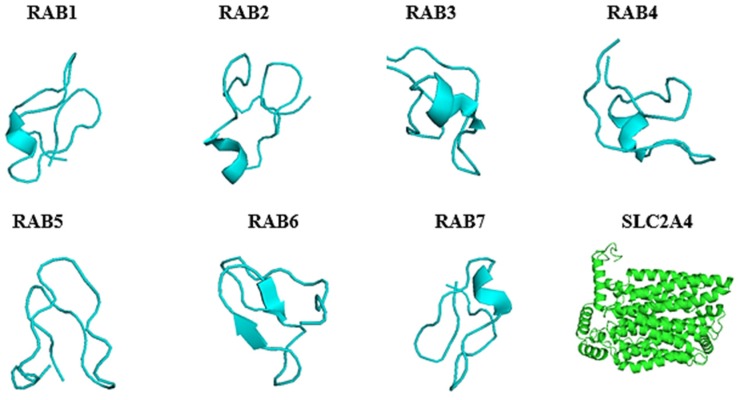
3D homology model of *Slc2a4* and putative anti-cancer AMPs using I-TASSER and visualized using PyMol. The structures in turquoise colour depicted the predicted 3D model of anti-cancer AMPs, and the structure in green colour represented the predicted 3D model of *Slc2a4*.

The 3D homology model structure of the putative anticancer AMPs and *Slc2a4* were analysed using I-TASSER and visualized using PyMol. As showed in (Figure 3) the I-TASSER results indicated that all the model structure of the putative anticancer peptide contained majorly β-strand which shows that the peptide can modulate and dictate the binding affinity or specificity to their receptor, and it can also help in more promiscuous ligand-protein or protein-protein contacts [25]. Furthermore, I-TASSER result showed that *Slc2a4* is made up of 21 helices 359 (70.5%) α-helix, 8 (1.6%) 3–10 helix, and 142 (27.9%) for others, making 509 residues in total. However, the 3D homology model contradicted the 2D structure, which calls for further investigations. As shown in Table 3, the confidence score (C-score), template modeling score (TM-score) and RMSD of the putative anticancer peptide and the protein 3D homologue model were of good model. The C-score which is the confidence score for evaluating the quality of a predicted model was above the limiting value of −1.5. Thus, all predicted peptides model was good model because C-score ranges from −5 to 2 [26,27,28]. Furthermore, the TM-scores were all above the limiting TM-score of >0.5 indicating that all the model are of correct topology and a TM-score < 0.17 indicates a random similarity [26,27,28]. All the built model has a good and considerable RMSD which ranges from 0.5–0.9 Å for putative anticancer AMPs and 6.4 ± 3.9 for *Slc2a4*.

### 2.5. The 3D Modelled Structure Validation

**Figure 4:**
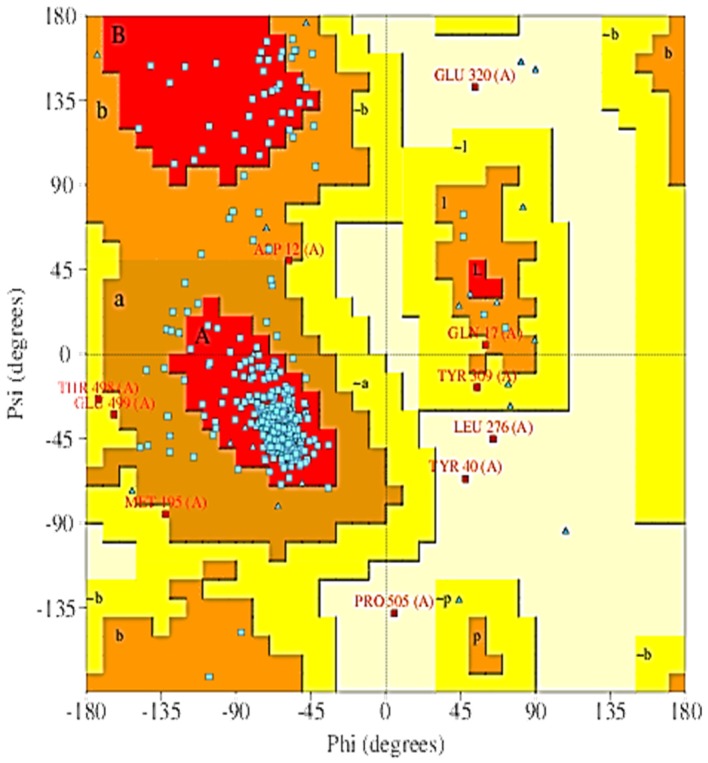
PROCHECK result of modelled *Slc2a4* using the generated model from i-TASSER. Residues in most favoured regions (A, B, L), Residues in additional allowed regions (a, b, l, p) and residues in generously allowed regions (~a, ~b ~l, ~p).

**Figure 5:**
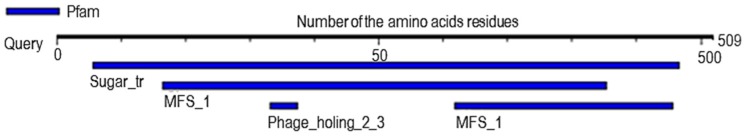
The predicted promising functional motifs present in *Slc2a4* generated by motif finder.

Thereafter, the quality of the model was evaluated and validated using PROCHECK, a program that relies on Ramachandran plot for structure verification [29]. As shown in Figure 4, results from the PROCHECK ascertained that the model has 87.4% residues in the most favoured regions, 10.5% residues in the additional allowed regions, 1.4% residues in the generously allowed regions and 0.7% in the disallowed regions. Therefore, the predicted model is considered to be of high quality because of the percentage distribution of the amino acid residues. The plot analysis is represented in Figure 5. Also, G-factor that provides a measure of how unusual or conversely how usual a given stereochemical property is [30] was also determined using this program. A G-factor of less than −0.5 is unusual and less than −1.0 indicates highly unusual. However, the generate G-factor for this model was −0.43 for dihedral angels, −0.07 for main chain covalent forces and −0.26 overall.

### 2.6. Analysis of Probable Functional Motifs Presents in Slc2a4 Protein

As shown in Table 4 and Figure 5, investigation of the functional probable motif ascertained that *Slc2a4* contains three functional motifs. Sugar_tr, the first motif, is located at position 28 to 483 in the amino acid sequence with an E-value of 1.8 × 10^−152^, MFS_1 located between position 82 to 427 in the amino acid sequence with E-value of 1.8 × 10^−15^ and position 309 to 478 in the sequence with E-value of 309–478 are the second motif, and lastly, Phage_holin_2_3 located between position 166 to 187 in the amino acid sequence with an E-value of 7.5 × 10^−3^ is the third motif.

### 2.7. The Evaluation and Recognition of Active Sites in Slc2a4 Protein

Assessment of the protein-ligand binding site prediction for structure-based biological function annotation of *Slc2a4* was done using COACH protein-ligand binding prediction server. The COACH server predicted all residues that involved in the formation of active site pockets for ligands, which are showed in Table 5. All the predicted ligands binding site by COACH server are convincing, because the C-scores ranging between 0 and 1, and scores close to zero represent random prediction while scores close to 1 indicates more reliable predictions [31].

### 2.8. The Protein-Peptide Interaction between the Putative Anticancer AMPs and Slc2a4 Protein

**Figure 6:**
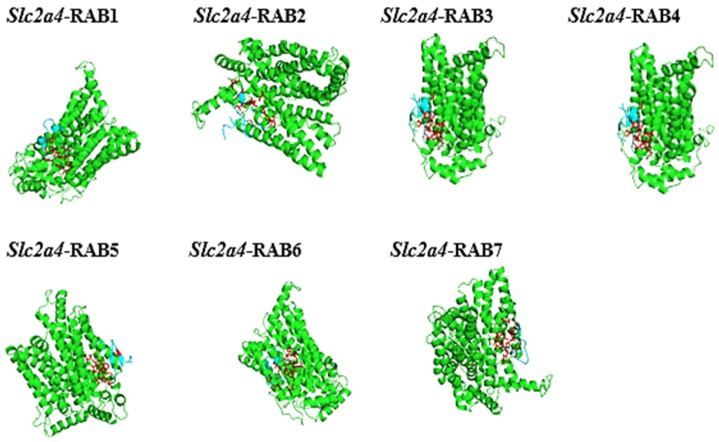
Interaction of putative anti-cancer AMPs with *Slc2a4*. The turquoise colours depicted the anti-cancer AMPs (RABs), green colours represented the *Slc2a4* and red colours shown the binding site.

The docking results of the structural complex between the putative anticancer AMPs and the *Slc2a4* protein were downloaded as PDB files, and they were all visualized using PyMol software. This is shown in Figure 6 in which the receptor (*Slc2a4*) as green surface and the ligand (AMPs) are shown in turquois blue.

## 3. Disccusion

Solute Carrier Family 2 Member 4 (*Slc2a4*), otherwise known as GLUT4, is a member of the glucose transporter family [32]. *Slc2a4* can serve as a potential biomarker for diverse kinds of malignant tumours such as; breast cancer, lung carcinoma, gastric cancer, and endometrial carcinoma [33,34]. Although, the exact mode of action between *Slc2a4* and cancer formation, progression and metastasis as not been fully understood. The uncontrolled growth and proliferation of cancer cells need a constant supply of metabolic energy, and glycolysis is one of the main biochemical process that characterised tumour cells, and the glycolytic breakdown of glucose is initiated by the transport of glucose, a rate-limiting process which is mediated by GLUT [35]. Moreover, an increase in GLUT upregulation in malignant cells has been linked with the overexpression of GLUT proteins, which supply steady metabolic energy to cancer cells [35]. Numerous research studies have hypothesis several ways in which *Slc2a4* results in cancer progression and metastasis one of which is the regulation of TRIM24–DDX58 axis that promote head and neck cancer (HNCC) metastasis [36]. Also, *Slc2a4* has been shown to participates actively in glucose uptake in androgen-insensitive than in androgen-sensitive in prostates cancer cells [35]. To the best of our knowledge, this study is novel and significant as it’s the first report on AMPs as inhibitors of *Slc2a4* for the treatment of cancer. Therefore, we have in this study analysed the *Slc2a4* protein sequence and designed seven putative anticancer AMPs as well as studied the protein-peptide interaction between the putative AMPs and the *Slc2a4* protein using several types of highly precise bioinformatics tools.

In *Slc2a4*, cysteine and tryptophan contributed less stability and folding state to the protein, due to the presents of their lesser amount in the protein. Cysteine contain reactive sulfhydryl group (-SH) that has ability to oxidize and form a disulphide bond (-S-S-) to a second cysteine [18]. Also, tryptophan contains an aromatic side chain that makes it able to interact with the membrane interface, stabilize the membrane protein, and contribute to hydrophobic mismatch response [37]. Thus, the stability of the *Slc2a4* protein might be due to the burial of polar groups and their hydrogen bonds [38] as well as the net charge and the ionization state of the individual amino acid residues [39]. The stability of *Slc2a4* protein may also be due to the presence of thousands of noncovalent bonds between amino acids and the availability of chemical forces between the protein and its immediate environment [40].

Hence, there are several ongoing searches for novel drugs that can serves as alternative medications in the treatment and management of cancer. Antimicrobial peptides have gained noticeable quality as possible sources of novel medicine in the treatment and control of diseases. This significant important of therapeutic applications of antimicrobials peptide in the treatment and control of diseases may be due to their size as well as their diverse activities and properties triggered toward diseases [17]. In this study, seven (7) putative anticancer AMPs were generated, with the majority having a Boman index less than or equal to one which indicates that the peptides will make good antimicrobial activity with minimal adverse effects. Also, the zero-net charge of some of the peptides may influences the binding capacity of those peptide to bind to the receptor. It has been shown that AMPs with Boman index less than zero (0) only possess antibacterial activity while AMPs with Boman index greater than zero have multifunctional hormone-like activities [41]. The 3D structure of the seven putative anticancer AMPs possess β-strand which can help in the folding complementation, insertion and intermolecular interactions between the hydrogen-bonding edges of β-sheets. This type of hydrogen bond interaction has been shown to contribute majorly to the fundamental form of biomolecular recognition, as well as, involvement in protein quaternary structure, protein and peptide aggregation and protein-protein interactions [42]. Therefore, the presence of β-sheet in AMPs make them to interact with another biomolecule; this biological importance exhibited by AMPs make it to have significant impact as potential targets for intervention in diseases. Furthermore, the 3D structure of *Slc2a4* protein was determined and both the 3D structure of the modelled putative anticancer AMPs and the *Slc2a4* protein are of good quality; when considering their C-score, TM-score and RMSD value. Likewise, the reliability and quality of the modelled *Slc2a4* protein was further evaluated using PROCHECK, which revealed that the protein 3D modelled structure is deemed to be of high quality because of the percentage distribution of the amino acid residues in the most favoured, allowed, disallowed, and generously allowed regions. Thereafter, three major probable functional motifs were predicted, and several ligands active sites presents in the *Slc2a4* protein was recognised.

Finally, the generated anticancer putative AMPs were used in this study to inhibits *Slc2a4*; an important protein that responsible for energy productions during angiogenesis in tumour formation and this can serve as another novel therapeutic agent for the treatment and management of cancer.

In this study, online protein-protein interaction tools PatchDock and FireDock server were used as docking tool for our putative anticancer AMPs (RAB1- RAB7) with *Slc2a4* protein. The generated 3D structure of the seven-novel anticancer putative AMPs serves as ligands, and each ligand were docked against the 3D structure of *Slc2a4* protein respectively. The docking runs for each of the parameter (AMP and *Slc2a4*) were done using PatchDock server and all the results generated by this server showed a very high binding affinity (score) which is greater than 8741 [43]. This indicates good binding solution for all the putative anticancer AMPs with RAB 1 having the highest binding affinity (score) as shown in Table 6. Furthermore, the generated results were refined using FireDock server, and all the results strongly supports the PatchDock score with a very good binding energy that is; the global energy in which RAB 5, RAB 4, and RAB 3 has less binding energy with −68.18, −55.01 and −53.99 kcal/mol, respectively. Altogether, the results reported in this study can usefully be employed for the rational design of novel, selective and potent *Slc2a4* inhibitors in the search for novel anti-cancer therapy with high efficacy.

## 4. Materials and Methods

### 4.1. Sequence Retrieval and Physicochemical Parameters Analysis of Slc2a4

The amino acid sequences of the protein *Slc2a4* were retrieved from the Universe Protein Resource (UniProt) (http://www.uniprot.org/), a database containing 80 million sequences [44]. The physicochemical parameters of the protein were generated from the ProtParam tools (http://web.expasy.org/protparam/) using the ExPAsy server. They reveal vital information about the protein structure and function. Knowing that the physicochemical parameters of a protein is of paramount importance, the following parameters were computed by the ProtParam tool: molecular weight, extinction coefficient, amino acid composition [45], estimated half-life and grand average of hydropathicity (GRAVY), theoretical pI [46], as well as aliphatic index [47] and instability index.

### 4.2. Secondary Structure Prediction

Prediction of the secondary structure (α-helices, β-sheets and random coils) of *Slc2a4* was done using PsiPred secondary structure prediction server (http://bioinf.cs.ucl.ac.uk/psipred). PSIPRED is a highly accurate tool for protein secondary structure prediction from its primary sequence, which integrates two feed-forward neural networks that perform an analysis on output obtained from PSI-BLAST (Position Specific Iterated-BLAST) [48].

### 4.3. Experimentally Validated Antimicrobial Peptides (AMPs) Data Assessment

Several experimentally validated anticancer antimicrobial peptides (AMPs) originating from plants were retrieved from different antimicrobial peptide databases that include Collection of Antimicrobial Peptides (CAMP) (http://www.camp.bicnirrh.res.in) and Data Repository of Antimicrobial Peptides (DRAMP) (http://dramp.cpu-bioinfor.org). Subsequently, the retrieved data was organised and curated to verify the authenticity of the experimentally validated anticancer AMPs. Thereafter, Cluster Database at High Identity with Tolerance (CD HIT) (http://www.bioinformatics.org/cd-hit) was used to remove the experimentally validated anticancer AMPs which are in duplicate.

### 4.4. Construction of AMPs Profile Using Hidden Markov Models

The list of the generated plants experimentally validated anticancer AMPs was divided into two portions, in which one-quarter of the retrieved data was utilised as the testing set while the three-quarters of the retrieved data was used as the training set. The Hidden Markov Models (HMMER) algorithm version 2.3.2 was used to create HMMs profile by utilizing training sets. Afterward, the testing set was used to ascertain the strength of the profile. The HMMER multiple modules was used in the construction of the AMPs profile.

Firstly, using clustalW as multiple alignment tool, a multiple sequence alignment was done for the sequences in the training set using the command line:

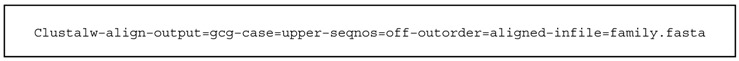


The result was saved as msf (gcg) format (family.msf). This aligned sequence was used for the second step, this step shows the common motifs within the model and it was achieved by inputting the command line:
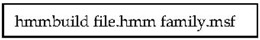



The sensitivity of the new profile HMM was enhanced by calibrating it using the command line:
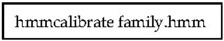



The resulting profile ‘family.hmm’ was employed in evaluating the performance of the profile by scanning it against AMPs in the testing set. The E-value threshold was set to 1% or 0.01% and the scanning was achieved using the command line:
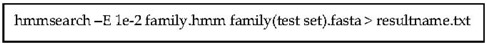



#### Discovery of Novel Putative Anticancer Amps from Genome Sequences

The ENSEMBL server (http://www.ensembl.org/index.html) and UNIPROT database (http://www.uniprot.org/) were visited to retrieve at least a thousand genome sequences in FASTA format. In order to identify the putative anti-cancer AMPs, the constructed profile was queried against all the genome sequences with an E-value cut-off of 0.01. This was achieved by utilizing the hmm search module of HMMER and the command line used is:
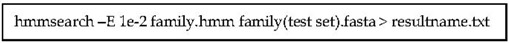



All identified peptides were considered to be putative anti-cancer AMPs.

### 4.5. Determination of the Physicochemical Parameters of the Putative Anti-Anticancer AMPs

Several physicochemical parameters of the putative anticancer AMPs were determined using the prediction interface of APD (http://aps.unmc.edu/AP/design/design_improve.php) and Bactibase (http://bactibase.pfba-lab-tun.org/physicochem), respectively. Some of the properties include molecular weight, the most abundant amino acids, net charge, isoelectric point, total hydrophobic ratio, Half-life, Boman index, and sequence similarity with other molecules and percentage (%).

### 4.6. Homology Modelling of the Three-Dimensional Structure of Slc2a4 and Putative Anti-Cancer AMPs

The 3D structure prediction of the seven putative anticancer AMPs and *Slc2a4* protein was done using I-TASSER server (http://zhanglab.ccmb.med.umich.edu/I-TASSER/) online database, the best results was obtained based on their E-values. The amino acid sequences were submitted to the online server of the I-TASSER and the result generated in PDB format was visualized using PyMol [49]. In predicting the 3D structure of *SLC2A4*, the protein with PDB ID: 4pypA (Crystal structure of the human glucose transporter GLUT1) was utilized as template for *de-novo* modeling of the *SLC2A4* protein, out of the 10 top templates chosen from the LOMETS threading programs. This is based on the highest significance in the threading alignments of the template with the query protein, which is measured by the Z-score; the difference between the average and raw scores in the unit of standard deviation [50].

### 4.7. Evaluation of the Generated Model

The stereochemical quality of the generated model was checked using a program called PROCHECK [51]. It verifies the quality of model by computing several parameters such as side chain conformations of protein structures as a function of atomic resolution, lengths, geometry of the hydrogen bonds, and angles and planarity of the peptide bonds [29].

### 4.8. Investigation of the Probable Functional Motif

MOTIF Finder (http://www.genome.jp/tools/motif/) an online search engine was used to ascertain the probable motifs present in the protein by analysis the amino acid sequences. A statistical expect value (E-value) cut off score is given because it shows the significance of the hit.

### 4.9. Active Site Analysis and Residues Recognition

The prediction of active site and possible ligand binding residues of *Slc2a4* was generated using COACH protein-ligand binding prediction server, a meta-server approach to protein-ligand binding site prediction (http://zhanglab.ccmb.med.umich.edu/COACH/). The complementary ligand binding site was predicted using COACH by matching the *Slc2a4* I-TASSER generated model with protein in the BioLiP protein function database. Also, the functional templates are detected and ranked by COACH using composite scoring function that is based on structure and sequence profile comparisons [26].

### 4.10. In Silico Protein-Protein Interaction Study

Having identified our putative anti-cancer AMPs, we decided to use PATCH Dock, an online protein-protein interaction server (https://bioinfo3d.cs.tau.ac.il/PatchDock), for performing the molecular docking. This server depends on shape complementarity of soft molecular surfaces to generate the best starting candidate solution [52]. The default clustering RMSD 4.0 Å was used and the complex type was chosen to be at protein-small ligand. The Connolly dot surface representation of the molecules into different component such as convex, concave, and flat patches were generated through PatchDock algorithm. Thereafter, the complementary patches were harmonized to form transformation candidates, which were later refined using FireDock server (http://bioinfo3d.cs.tau.ac.il/FireDock/), an online tool that optimised, refined, reshuffled, and rescored the side chains interface of the top 10 candidate solutions. It also amends the orientation of the relative molecules by confining the flexibility to the side-chains of the interacting surface and allow the movements of small rigid-body [53]. Therefore, the interactions and binding observed in the docked conformations in PDB format were visually using the PyMol software [49].

## Figures and Tables

**Table 1:** Physicochemical parameters of *Slc2a4*.

ProtParam Parameters	Values
Number of amino acids	509
Molecular weight	54,895.50 Da
Theoretical Pi	6.86
Atomic composition	Carbon C: 2530 Hydrogen H: 4009 Nitrogen N: 641 Oxygen O: 686 Sulfur S: 16
Formula	C_2530_H_4009_N_641_O_686_S_16_
Number of negatively charged residues	33
Number of positively charged residues	33
Extinction coefficient	59,610 Abs 0.1% (=1 g/L) 1.086, assuming all pairs of Cys residues form cystines 59,360 Abs 0.1% (=1 g/L) 1.081, assuming all Cys residues are reduced
Total number of atoms	7882
Estimated Half-life	30 h (mammalian reticulocytes, in vitro). >20 h (yeast, in vivo). >10 h (Escherichia coli, in vivo).
Aliphatic index	118.61
Grand average of hydropathicity (GRAVY)	0.556
Instability index	39.83

**Table 2:** Physicochemical properties for the seven putative anti-cancer antimicrobial peptides (AMPs).

Putative AMPs	Mass (Da)	Most Common Amino Acids	Isoelectric Point	Net Charge	Total Hydrophobic Ratio (%)	Bosman Index (kcal/mol)	Half-Life (h)	Sequence Similarity with Other Molecules and Percentage (%)
RAB 1	3038.03	Cys: 20.69	6.03	0	51	0.18	30	AP02325: 75
RAB 2	3091.14	Ile: 16.67	6.03	0	50	−0.11	30	AP01777: 80.64
RAB 3	3313.15	Ile/Val: 12.9	7.76	2	51	0.48	30	AP01990: 74.19
RAB 4	3245.94	Val: 12.9	5.53	0	51	0.56	30	AP01062: 77.41
RAB 5	3156.35	Cys: 21.43	4.31	−1	46	1.00	30	AP00274: 59.37
RAB 6	2930.31	Cys: 21.43	3.85	−1	60	−1.00	30	AP01777: 56.66
RAB 7	3197.90	Cys: 20.69	8.38	3	51	3	30	AP02661: 61.29

**Table 3:** Quality evaluation scores of the predicted 3D structures by I-TASSER.

Putative Anti-Cancer AMPs	C-Score	Exp. TM Score	Exp. RMSD (Å)
RAB 1	0.81	0.82 ± 0.08	0.5 ± 0.5
RAB 2	0.71	0.87 ± 0.09	0.5 ± 0.5
RAB 3	0.70	0.81 ± 0.09	0.6 ± 0.6
RAB 4	0.71	0.81 ± 0.09	0.6 ± 0.6
RAB 5	0.69	0.81 ± 0.09	0.5 ± 0.5
RAB 6	0.44	0.77 ± 0.10	0.9 ± 0.9
RAB 7	0.67	0.80 ± 0.09	0.6 ± 0.6
*Slc2a4*	0.42	0.77 ± 0.10	6.4 ± 3.9

**Table 4:** Motif.

S/N	Pfam ID	Position	E-Value	Description
1	Sugar_tr	28–483	1.8 × 10^−152^	Sugar (and other) transporter
2	MFS_1	82–427 309–478	1.8 × 10^−15^ 4 × 10^−3^	Major Facilitator Superfamily
3	Phage_holin_2_3	166–187	7.5 × 10^−2^	Bacteriophage holin family HP1

**Table 5:** Predicted ligand binding sites in the *Slc2a4* using the homologue model generated by I-TASSER.

Name of Server	Name of Ligand	Residue Number	C-Score
**COACH SERVER**	Maltose (MAL)	42, 46, 177, 180, 181, 184, 298, 299, 304, 333, 395, 396, 404, 431	0.19
	Cytochalasin B (5RH)	96, 153, 177, 180, 298, 304, 400, 424, 427, 428, 431	0.10
	Cholesterol hemisuccinate (Y01)	97, 275, 292, 296, 300, 426, 429, 434	0.09
	(2~{S})-3-(2-bromophenyl)-2-[2-(4-methoxyphenyl)ethanoylamino]-~{N}-[(1~{S})-1-phenylethyl] propenamide (5RF)	38, 96, 153, 176, 298, 395, 396, 420, 424, 427, 428	0.05
	Octyl Glucose Neopentyl Glycol (37X)	22, 23, 26, 218, 219, 222, 246	0.03
	(2S)-2,3Dihydroxypropyl (7Z)-pentadec-7-enoate (78M)	90, 91, 93, 94, 142, 426, 427	0.02
	Octyl Glucose Neopentyl Glycol (37X)	94, 97, 98, 101, 118, 143	0.02
	methyl-α-d-glucopyranoside (GYP)	296, 430, 433, 434, 452, 453, 456, 457	0.02
	N-[(1R)-1-phosphonoethyl]-l-alaninamide (AFS)	39, 43, 177, 180, 181, 184, 329, 333	0.02
	M-cresol (CRS)	306, 309	-
**TM-Site Results**	Maltose (MAL) (2), β-d-glucose (BGC) (2), 6-bromo-6-deoxy-β-d-glucopyranose (6BG) (1)	42, 46, 177, 180, 181, 184, 298, 299, 304, 333, 395, 396, 404, 431	0.4
	5RH (1), (2~{S})-3-(2-bromophenyl)-2-[2-(4-methoxyphenyl)ethanoylamino]-~{N}-[(1~{S})-1-phenylethyl] propenamide (5RF)(1), (2~{S})-3-(4-fluorophenyl)-2-[2-(3-hydroxyphenyl)ethanoylamino]-~{N}-[(1~{S})-1-phenylethyl]propenamide(5RE(1)	96, 153, 177, 180, 298, 304, 400, 424, 427, 428, 431	0.2
	Cholesterol hemisuccinate (Y01) (2)	97, 275, 292, 296, 300, 426, 429, 434	0.1
	Octyl Glucose Neopentyl Glycol (37X) (1)	22, 23, 26, 218, 219, 222, 246	0.1
**S-Site Results**	2′-deoxycytidine-5′-monophosphate (DCM) (1), 2′-deoxyuridine 5′-monophosphate (UMP) (1)	349, 350	0.16
	M-cresol (CRS) (1), adenosine-5′-phosphosulfate (ADX) (1)	304, 306, 309	0.15
	magnesium ion (Mg^2+^) (1), calcium ion (Ca^2+^) (1)	224, 225, 226, 228	0.15
	magnesium ion (Mg^2+^) (1)	162, 165	0.14
	l-α-glycerophosphorylethanol amine (GPE) (1), Fe (II) ion (Fe^2+^) (1)	239, 299, 302, 303, 400, 409	0.14

**Table 6:** PatchDock and FireDock results for each AMPs, with the binding affinity and binding energy for the solution in kcal/mol.

Molecule (AMPs)	Binding Affinity for Geometry Scores	Global Energy (Binding Energy for the Solution) (kcal/mol)
RAB 1	12,392	−39.13
RAB 2	11,528	−36.74
RAB 3	10,154	−53.99
RAB 4	10,768	−55.01
RAB 5	11,146	−68.19
RAB 6	10,556	−25.85
RAB 7	11,558	−36.88
